# Benign yet challenging: a case of adult choroid plexus papilloma

**DOI:** 10.11604/pamj.2025.52.7.48595

**Published:** 2025-09-04

**Authors:** Nirlipta Swain, Jayashree Bhawani

**Affiliations:** 1 Department of Pathology, Jawaharlal Nehru Medical College, Datta Meghe Institute of Higher Education and Research, Sawangi (Meghe), Wardha, Maharashtra, India

**Keywords:** Headache, fibrovascular, columnar, hydrocephalus, cerebrospinal fluid

## Image in medicine

Choroid plexus papilloma (CPP) is a rare, benign epithelial tumor of the central nervous system, accounting for 0.4–0.6% of brain tumors. Arising from the choroid plexus, which produces cerebrospinal fluid (CSF), CPPs usually grow slowly and lack metastatic potential. They are more frequent in children, typically in the supratentorial region, while in adults they occur predominantly in the infratentorial compartment. Clinically, CPPs often cause hydrocephalus and raised intracranial pressure due to CSF overproduction or obstruction, presenting as headache, nausea, vomiting, or altered sensorium. MRI is the preferred diagnostic tool, aiding assessment of tumor size, vascularity, and relation to adjacent structures. Surgical excision remains the treatment of choice and generally ensures a favourable prognosis. We present the case of a 50-year-old male with a one-month history of persistent headache and dizziness, without seizures, vomiting, or neurological deficits. Examination and laboratory results were normal. MRI suggested a vascular malformation in the right parietal lobe extending into the choroid plexus. The patient underwent uneventful surgical excision under general anaesthesia. Grossly, the specimen was an irregular, brownish-black fragment with a bosselated surface. Microscopy revealed papillary fronds with delicate fibrovascular cores lined by low columnar epithelium, scant stroma, and dystrophic calcification features diagnostic of CPP. Unlike normal choroid plexus tissue with its “cobblestone” pattern, CPP lacked apical intercellular separations. According to the World Health Organization (WHO) classification, CPP is grade I, atypical CPP grade II, and choroid plexus carcinoma grade III. This case underscores the importance of including CPP in the differential diagnosis of intraventricular lesions in adults, despite its rarity. The patient was discharged in stable condition and advised regular neurosurgical follow-up. This case emphasizes considering CPP in adult intraventricular or periventricular lesions despite its rarity.

**Figure 1:**
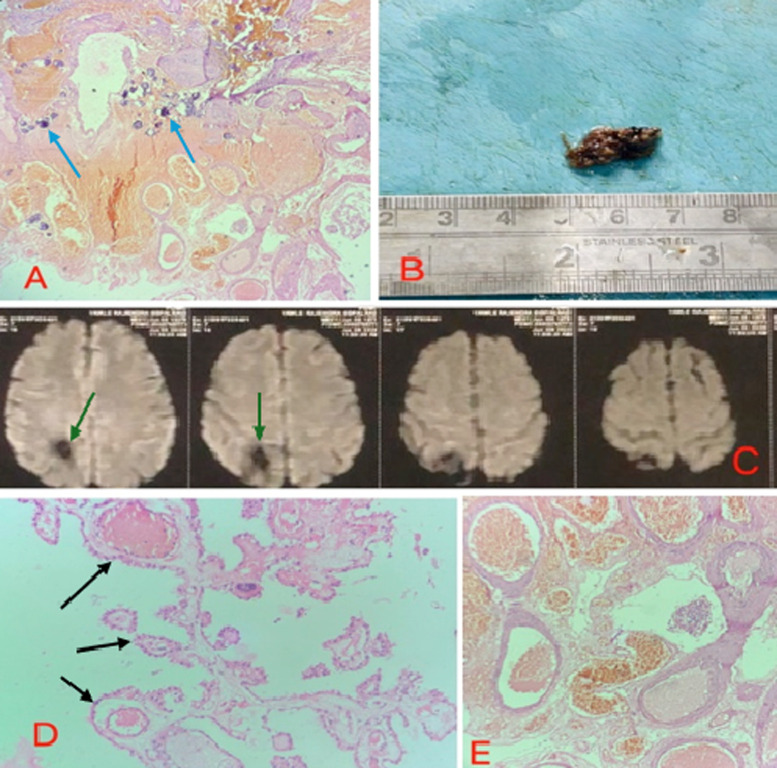
A) microscopy revealing areas of dystrophic calcification (blue arrow) 20x H&E; B) gross picture of the excised specimen of cavernoma; C) MRI showing vascular abnormality in right parietal lobe extending to right side choroid plexus (green arrow); D) microscopy revealing fine fibrovascular fronds lined by low columnar epithelium (black arrow) with scant stromal tissue 20x H&E; E) microscopic representation of the tumor at 40x H&E

